# Effect of Du-Moxibustion Combined With Spine-Pinching Therapy on Cognitive Frailty in Older Adults With Prefrailty: Protocol for a Randomized Controlled Trial

**DOI:** 10.2196/82683

**Published:** 2026-02-10

**Authors:** Suzhen Liu, Mengxia Qi, Hua-Fang Li, Ziqiu Ye, Yu Xu, Xiangying Yang

**Affiliations:** 1 The Geriatrics Department, Affiliated Hangzhou First People's Hospital, School of Medicine, Westlake University, No. 261 Huansha Road, Shangcheng District, Hangzhou, Zhejiang, 310006, China, 86 13732200256, 86 56006871; 2 School of Nursing, Zhejiang Chinese Medical University, Hangzhou, Zhejiang, China; 3 The Fourth Clinical Medical College, Zhejiang Chinese Medical University, Hangzhou, Zhejiang, China

**Keywords:** cognitive frailty, Du-moxibustion, older adults, prefrailty, randomized controlled trial, RCT, spine-pinching therapy

## Abstract

**Background:**

Cognitive frailty (CF), characterized by the coexistence of mild cognitive impairment and physical frailty in the absence of dementia or other neurodegenerative diseases, is a significant risk factor for dementia and functional decline in older adults. Although Du-moxibustion has shown potential benefits in improving CF, the effect of spine-pinching therapy remains underexplored.

**Objective:**

This study aims to evaluate, for the first time, the efficacy and safety of Du-moxibustion combined with spine-pinching therapy in older adults with prefrailty and CF.

**Methods:**

This is a prospective, single-center, randomized, single-blind, 4-arm parallel controlled trial. A total of 156 older adults with prefrailty and CF will be recruited and randomly assigned to 1 of 4 groups: routine care group, Du-moxibustion group, spine-pinching group, or combined intervention group. The intervention will last for 8 weeks. The primary outcome is the change in Montreal Cognitive Assessment score. Secondary outcomes include the Fried frailty phenotype, Barthel Index (activities of daily living), 15-item Geriatric Depression Scale, 36-item Short Form Health Survey, global frailty status, and traditional Chinese medicine syndrome scores. Outcome assessments will be performed at baseline (wk 0), midintervention (wk 6), postintervention (wk 8), and follow-up (wk 12).

**Results:**

Recruitment for this study is scheduled to commence in March 2026 and will end in June 2027 (recruitment and intervention). All follow-up and data collection activities will be finalized by October 2027. Results are anticipated to be completed in the first quarter of 2028.

**Conclusions:**

This study is expected to provide high-quality evidence for the clinical efficacy of Du-moxibustion combined with spine-pinching therapy in managing CF and contribute to the integration of traditional Chinese medicine external therapies in the promotion of healthy aging. Although the single-blind design may introduce expectancy bias, strict randomization procedures and standardized interventions will enhance the reliability and scientific rigor of the results.

## Introduction

Cognitive frailty (CF), defined as the coexistence of physical frailty and mild cognitive impairment (MCI) in the absence of dementia, has been increasingly recognized as a critical geriatric syndrome associated with accelerated functional decline, disability, hospitalization, and mortality [1]. Recent epidemiological studies report that the prevalence of CF ranges from 1% to 12% in community-dwelling older adults and is substantially higher among hospitalized populations [2]. With the rapid demographic shift in China, the number of older adults is projected to exceed 280 million by 2030, suggesting that the burden of CF will continue to rise [3].

Prefrailty, a transitional and potentially reversible stage within the frailty continuum, affects more than half of older adults and represents an important window for targeted preventive intervention [4,5]. However, evidence indicates that prefrailty is frequently underrecognized and undertreated in clinical settings, resulting in missed opportunities to delay or reverse progression toward frailty and cognitive decline [6,7]. Current Western medicine approaches for managing CF—including pharmacological treatments such as cholinesterase inhibitors and nonpharmacological strategies such as cognitive training, physical exercise, and psychosocial support—offer limited benefits on combined cognitive and physical outcomes, and pharmacological approaches often carry risks of adverse events in older adults [8,9]. These limitations highlight the need for safe, multidimensional, and holistic interventions specifically tailored to the population with prefrailty.

Traditional Chinese medicine (TCM) emphasizes individualized, syndrome-based treatment and has shown potential in improving both cognitive performance and frailty-related physical deficits. External TCM therapies such as Du-moxibustion and spine-pinching (niefu) therapy are believed, according to TCM theory, to regulate physiological balance, support spleen-kidney function, and enhance vitality. Emerging evidence suggests that Du-moxibustion may improve cognitive function, physical frailty symptoms, gait performance, and inflammatory biomarkers in older adults with frailty or cognitive impairment [10-12]. Spine-pinching therapy, widely used in TCM rehabilitation to modulate meridian pathways and autonomic function, has demonstrated preliminary benefits in improving gastrointestinal function, physical vitality, and neurocognitive-related symptoms [13,14]. Nevertheless, despite their complementary theoretical basis, no randomized controlled trial (RCT) has evaluated the combined effects of Du-moxibustion and spine-pinching therapy in older adults with prefrailty-related CF.

To address this gap, this study will conduct an RCT to evaluate the efficacy and safety of Du-moxibustion combined with spine-pinching therapy in older adults diagnosed with prefrailty and CF, based on both Western diagnostic criteria and a specific TCM syndrome pattern (“spleen–kidney deficiency with Du meridian obstruction”). This trial aims to provide high-quality evidence supporting an integrative, multidimensional intervention strategy for improving cognition, frailty status, emotional well-being, and quality of life in this growing vulnerable population.

## Methods

### Ethical Considerations

This study has been approved by the ethics committee of Hangzhou First People’s Hospital (approval 2025ZN211-1), in compliance with the Declaration of Helsinki (Edinburgh 2000 Amendment) and medical ethical requirements. Concurrently, this trial is registered with the International Traditional Medicine Clinical Trial Registry under the registration number ITMCTR2025001589. Participants received comprehensive study information, including the study’s purpose, potential risks (eg, burns or emotional distress), benefits, and their right to withdraw at any time, and provided written informed consent prior to screening. Serious adverse events will be reported to the ethics committee within 24 hours and monitored by the Data and Safety Monitoring Committee (DSMC) throughout the 8-week intervention and 12-week follow-up. Patients or their legal representatives were informed in an accessible language about the study objectives, methods, and procedures. During the preparation of this study, generative artificial intelligence tools were used to assist with translation and linguistic refinement. Results will be submitted to peer-reviewed SCI (Science Citation Index) journals and presented at conferences on geriatrics, TCM, and nursing. Anonymized data will be uploaded to an open-access repository for validation and future research.

### Study Design

This study is designed as a prospective, single-center, randomized, single-blind, 4-armed parallel controlled clinical trial. A total of 160 eligible patients will be randomly assigned in a 1:1:1:1 ratio to one of four groups via a centralized randomization system: routine care group, Du-moxibustion group, spine-pinching group, and combined intervention group (Du-moxibustion+spine-pinching). The intervention will last for 8 weeks. The routine care group will receive health education once per week, while the other 3 groups will receive treatment twice per week: Du-moxibustion (30 min/session), spine-pinching (15‐20 min/session), and combined therapy (45‐50 min/session). Outcomes will be assessed at baseline, midintervention, postintervention, and follow-up.

### Participants

#### Source of Participants

Participants will be recruited from the geriatrics and neurology outpatient clinics of Hangzhou First People’s Hospital between March 2026 and June 2027. Recruitment will be conducted through outpatient screening and community-based advertisements.

#### Inclusion Criteria

Participants must meet all of the inclusion criteria summarized in Textbox 1.

Textbox 1. **Inclusion criteria**

- Aged ≥60 years
- Fried frailty phenotype score between 1 and 2, indicating a prefrail state
- Diagnosed with cognitive frailty, as indicated by a Montreal Cognitive Assessment score of <26
- Diagnosed with the traditional Chinese medicine (TCM) syndrome of “spleen-kidney deficiency with Du meridian obstruction,” according to the Standards for TCM Disease and Syndrome Diagnosis and Efficacy Evaluation [15] (see “Diagnostic Criteria for CF” section for diagnostic criteria: ≥2 primary symptoms, ≥2 secondary symptoms, and corresponding tongue and pulse signs)
- Able and willing to provide written informed consent and comply with the study protocol

#### Diagnostic Criteria for CF

Participants will be screened based on both Western medicine and TCM diagnostic criteria.

##### Western Medicine Diagnostic Criteria

Based on the definition by the International Academy on Nutrition and Aging or the International Association of Gerontology and Geriatrics [1], the diagnostic criteria for CF include: (1) patient or family member reports subjective cognitive decline; (2) no diagnosis of dementia, with a Clinical Dementia Rating score of 0.5; and (3) Mini–Mental State Examination score ≤27 or MoCA score <26.

These criteria align with the international consensus definition of CF—coexistence of physical frailty and MCI without dementia.

##### TCM Diagnostic Criteria

According to the Standards for TCM Disease and Syndrome Diagnosis and Efficacy Evaluation [15], patients must meet the following criteria for the TCM syndrome of “spleen-kidney deficiency with Du meridian obstruction”:

1. Primary symptoms: fatigue, soreness, and weakness of the waist and knees, poor appetite, and insomnia;
2. Secondary symptoms: dizziness, abdominal distension, lower back pain, abdominal cold pain, tinnitus, edema, pale or flushed complexion, aversion to cold or 5-center heat, spontaneous or night sweating, dry mouth or thirst, frequent urination or nocturia, and loose or dry stools;
3. Tongue and pulse signs: pale or swollen tongue, or red tongue with scant coating; weak, deep-slow, or thin-rapid pulse.

A diagnosis can be made when the patient presents with at least 2 primary symptoms, 2 secondary symptoms, and characteristic tongue and pulse signs.

### Exclusion Criteria

Participants will be excluded if they meet any of the criteria summarized in Textbox 2.

Textbox 2. **Exclusion criteria**

- Diagnosed with Alzheimer disease or other forms of dementia
- Suffering from severe cardiovascular, cerebrovascular, hepatic, renal, or psychiatric disorders
- Received Du-moxibustion, spine-pinching, or other external traditional Chinese medicine therapies in the past 6 months
- Allergic to moxa, ginger, or other topical substances used in the intervention, or have skin ulcers, infections, or other conditions that contraindicate treatment
- Unable to maintain prone or lateral positions due to severe spinal deformities, arthritis, or other conditions
- Participated in other exercise, cognitive training, or intervention studies within the past 3 months

### Criteria for Withdrawal or Discontinuation

Participants will be withdrawn or excluded from analysis under the following circumstances: (1) voluntary withdrawal or loss to follow-up before completing the intervention, (2) serious adverse events or complications during the study, (3) poor compliance leading to discontinuation of treatment, and (4) development of severe illness during the study period that requires termination of participation.

### Sample Size Calculation

This study uses the MoCA score as the sole primary outcome measure, focusing specifically on the change from baseline to week 8. The primary comparison of interest is the difference in therapeutic efficacy between the combined intervention group and the routine care group.

Based on previous literature concerning nonpharmacological TCM interventions for cardiovascular and cognitive conditions, we hypothesize that the improvement in MoCA scores in the combined intervention group after 8 weeks will exceed that of the routine care group by a clinically meaningful difference (△µ) of approximately 3 points. We acknowledge that the estimated effect size (standardized mean difference ≈0.7-0.9) cited in similar studies may appear optimistic. However, this anticipated 3-point improvement is supported by the moderate-to-strong effect trend consistently reported in meta-analyses of comparable TCM external therapies, justifying its use for the primary calculation.

Assuming an SD (σ) of approximately 4, a one-way ANOVA was used to estimate the required effective sample size. The significance level (α) was set at .05, and statistical power (1–β) at 0.80. The initial calculation indicated a required effective sample size of 33 participants per group.

Considering a potential dropout rate of 15%, the required number of participants to be recruited per group is calculated as approximately 33/(1‐0.15)=38.82. The final sample size was therefore rounded up to 39 participants per group to ensure adequate retention and statistical power, resulting in a total sample size of 156 participants.

### Randomization, Allocation, and Blinding

#### Randomization and Allocation Concealment

Randomization will be conducted independently by a research assistant at the study center. Eligible participants will be randomly assigned in a 1:1:1:1 ratio to the routine care group, Du-moxibustion group, spine-pinching group, or combined intervention group. An independent statistician will generate a stratified random number list using SAS 9.3 software (SAS Institute Inc), stratified by age and MoCA score, with a block size of 6 or 9. Randomization will be implemented through an online central randomization system. Each participant will be assigned a unique identification number to ensure allocation concealment and prevent selection bias.

#### Blinding

This study uses a single-blind design. Outcome assessors and data analysts will be blinded to group allocation, whereas intervention practitioners cannot be blinded due to the nature of Du-moxibustion and spine-pinching procedures. Participants will be informed they are receiving treatment but will not be told the specific group or intervention type (eg, Du-moxibustion or spine-pinching) to minimize expectation bias. All assessors will undergo standardized training in MoCA and Fried phenotype scoring to ensure objective and consistent evaluations. Statistical analysis will be performed by an independent third party, and raw data will be anonymized. In the case of emergencies (eg, serious adverse events), unblinding may occur, with reasons, timing, and actions recorded in the case report form (CRF). Final unblinding will occur after study completion and data analysis.

### Intervention Methods

All participants will receive standardized routine care as a basic treatment. Interventions will be administered by licensed TCM practitioners or trained technicians according to standardized procedures to ensure consistency. During the intervention period, participants will be instructed to avoid receiving any additional external TCM treatments or other therapies that may affect CF.

### Routine Care Group

Participants in the control group will receive 30-minute health education sessions twice per week, conducted by registered nurses in classrooms or consultation rooms. Education will be delivered using Microsoft PowerPoint presentations, video demonstrations, and interactive Q&A sessions. Printed health education manuals will also be provided. The curriculum includes:

1. Dietary guidance: low-salt, low-fat diet with balanced nutrition (eg, omega-3-rich fish, fruits, and vegetables);
2. Exercise advice: moderate aerobic exercise (eg, 30 min of brisk walking or Tai Chi daily);
3. Cognitive health strategies: memory training techniques (eg, associative memory and number games) and psychological health management.
4. Frequency and duration: twice per week for 8 consecutive weeks (total of 16 sessions).
5. Safety: any psychological or emotional discomfort (eg, anxiety) reported after sessions will be recorded. No invasive procedures are involved.

### Du-Moxibustion Group

**Figure 1.**
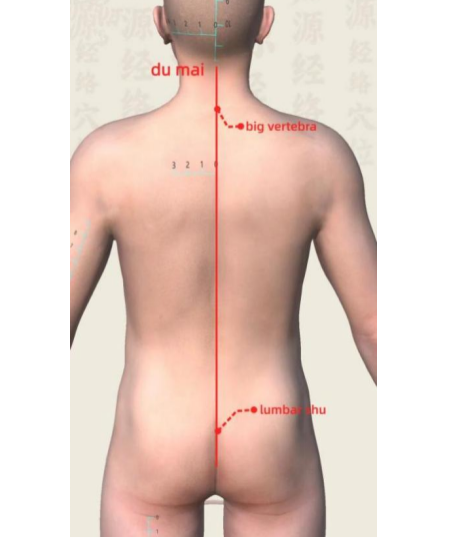
Acupoints and treatment area for Du-moxibustion along the Du meridian (GV14–GV2).

Details pertaining to the participants in the Du-moxibustion group are as follows:

- Treatment site: this implies the Du meridian line, from Dazhui (GV14) to Yaoshu (GV2), covering thoracic vertebrae T1 to lumbar vertebrae L5, as illustrated in Figure 1.
- Materials: these include a herbal powder (equal parts of astragalus, codonopsis, chuanxiong, and carthamus), fresh ginger paste (thickness: 1.5 cm), mulberry paper, moxa cones (1.5 cm diameter, approximately 2 g each), alcohol swabs, and an ignition device.
- Procedure: these include (1) the participant lying in the prone position; (2) the back is cleaned and dried; (3) the herbal powder is applied along the Du meridian, covered with mulberry paper; (4) a 1.5 centimeter layer of ginger paste is spread over the area; (5) Moxa cones are placed in shallow depressions formed in the ginger layer; (6) the cones are ignited and burned for approximately 30 minutes, and (7) the residues are cleaned and the skin is inspected for irritation or burns.
- Environment: the treatment room must be well-ventilated and equipped with smoke evacuation systems.
- Frequency and duration: the frequency is twice per week for 8 consecutive weeks (total of 16 sessions). The session duration of Du-moxibustion was set at 30 minutes based on previous clinical trials, in which moxibustion treatments typically lasted 20 to 30 minutes, ensuring both sufficient stimulation and patient safety [16].
- Safety monitoring: skin reactions (eg, redness and burns) will be observed after each session. Cold compresses or suspension of treatment will be applied if necessary. All findings will be recorded in the CRF.

### Spine-Pinching Group

**Figure 2.**
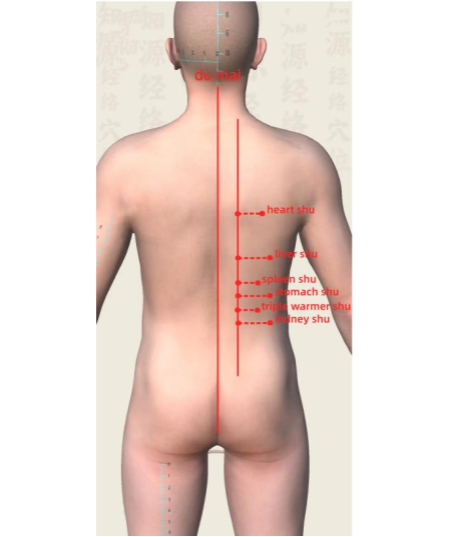
Acupoint locations used in spine-pinching therapy.

**Figure 3.**
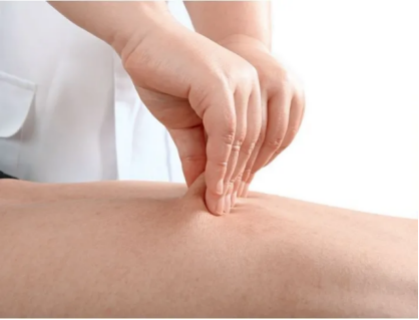
Manipulation technique of spine-pinching therapy.

Details pertaining to the participants in the spine-pinching group are as follows:

- Position: the position should be prone, with the back exposed.
- Method: starting from the sacrococcygeal region along the midline of the spine, pinch the skin and move upward toward the seventh cervical vertebra. The procedure is performed along 3 lines centered on the spine (left bladder meridian, right bladder meridian, and Du meridian). For each line, perform 3 pinches, followed by lifting (pinch 3 times and lift once) for 4 to 5 cycles. Acupressure is applied to the Xinshu (HT7), Shenshu (KI3), Pishu (SP3), Weishu (ST25), Ganshu (LV3), and Sanjiaoshu (SJ5) points (1 min per acupoint). The acupoint locations are shown in Figure 2, and the manipulation technique is illustrated in Figure 3. The total treatment duration is 15 to 20 minutes.
- Frequency and duration: the frequency is twice per week for 8 weeks, totaling 16 sessions.
- Safety: after each intervention, skin tenderness or muscle discomfort will be monitored and recorded in the CRF.

### Combined Intervention Group

Details pertaining to the participants in the combined intervention group are as follows:

- Intervention order: following the “first unblock, then replenish” principle, spine-pinching therapy is performed first (to unblock meridians and regulate qi and blood), followed by Du-moxibustion (to warm and regulate the Du meridian and tonify the kidney and marrow).
- Procedure: the spine-pinching procedure is the same as described in the “Spine-Pinching Group” section, and the Du-moxibustion procedure is the same as described in the “Du-Moxibustion Group” section. The total treatment time is 45 to 50 minutes.
- Environment: the treatment room should have proper ventilation and be equipped with smoke-extraction devices.
- Frequency and duration: the frequency is twice per week for 8 weeks, totaling 16 sessions.
- Safety: after each intervention, skin redness, burns, or tenderness will be monitored. If necessary, cold compresses or suspension of treatment will be applied, and the results will be recorded in the CRF.

### Intervention Adherence Management

A dedicated team member will record the execution of interventions and patient feedback. Prior to each session, the research nurse will inform, supervise, and reassure the participants. If a participant misses 2 consecutive sessions or completes less than 80% of the interventions, the reasons will be analyzed, and a decision will be made regarding their inclusion in the final analysis.

### Outcome Measures

#### Overview

During the screening phase, all participants will complete a questionnaire collecting demographic data such as gender, age, nationality, education level, occupation, marital status, as well as disease-related information such as onset time and duration. Prior to randomization, baseline measurements of MoCA and the Fried frailty phenotype will be taken. Clinical evaluations of both primary and secondary outcomes will be conducted at baseline (week 0), midintervention (week 6), postintervention (week 8), and follow-up (week 12). The timeline of the study is depicted in Table 1.

**Table 1.** SPIRIT (Standard Protocol Items: Recommendations for Interventional Trials) guidelines for the schedule of enrollment, interventions, and assessments.

	Baseline		Treatment phase			Baseline
	Week –2～0		Week 0	Week 6	Week 8	Week 12
Ethics and informed consent						
Ethics committee approval	✓					
Signature of informed consent			✓			
Screening and randomization						
Inclusion or exclusion criteria assessment	✓					
Random grouping			✓			
Allocation hiding			✓			
Pretest						
4 cases of pretest	✓					
Intervention						
Conventional control group (2 sessions/week, 30 min each)			✓	✓	✓	
Du-moxibustion group (2 sessions/week, 30 min each)			✓	✓	✓	
Spine-pinching group (2 sessions/week, 15‐20 min each)			✓	✓	✓	
Combined intervention group			✓	✓	✓	
Outcome assessment (4 sessions/week total)						
MoCA^a^			✓	✓	✓	✓
Fried			✓	✓	✓	✓
Barthel			✓	✓	✓	✓
SF-36^b^			✓	✓	✓	✓
GDS-15^c^			✓	✓	✓	✓
Traditional Chinese medicine syndrome scores			✓	✓	✓	✓
Safety control						
Adverse event monitoring			✓	✓	✓	✓
Serious adverse event report			✓	✓	✓	✓
Quality control						
Research personnel training	✓					
Compliance management			✓	✓	✓	

a MoCA: Montreal Cognitive Assessment.

b SF-36: 36-item Short Form Health Survey.

c GDS-15: 15-item Geriatric Depression Scale.

#### Primary Outcome Measures: Cognitive Function

Cognitive function will be assessed using the Chinese version of the MoCA. The total score ranges from 0 to 30, with higher scores indicating better cognitive function. The primary outcome is the change in MoCA scores from baseline. Evaluations will take place at weeks 0, 6, 8, and 12.

#### Secondary Outcome Measures

##### Physical Frailty

Physical frailty will be assessed using the Fried Frailty Phenotype Scale (0‐5 points). A score of 1 to 2 indicates prefrailty, and ≥3 indicates frailty. The scale includes measures of weight loss, fatigue, grip strength (measured using a standard dynamometer), walking speed (measured by a 4-m walking test), and physical activity reduction. Evaluations will be conducted by assessors at weeks 0, 6, 8, and 12. The main focus will be to observe whether participants transition from “prefrailty” to “nonfrailty.”

##### Activities of Daily Living

Activities of daily living will be assessed using the Barthel Index (0‐100 points, with higher scores indicating better function). The index includes 10 items related to basic daily activities such as feeding, bathing, and dressing. Assessments will be made at weeks 0, 6, 8, and 12.

##### Quality of Life

Quality of life will be measured using the Short Form Health Survey, self-administered by participants. It includes 8 dimensions, with scores ranging from 0 to 100. The results are summarized into 2 components: physical health component summary and mental health component summary, with higher scores indicating better quality of life. Evaluations will occur at weeks 0, 6, 8, and 12.

##### Depression Status

Depression will be assessed using the 15-item Geriatric Depression Scale, with a total score ranging from 0 to 15. A score ≥5 suggests the presence of depressive symptoms, and higher scores indicate greater depression severity. The scale will be administered through an interview conducted by an assessor. Evaluations will occur at weeks 0, 6, 8, and 12.

##### TCM Syndrome Score

The TCM syndrome score will be based on the syndrome differentiation criteria for “spleen-kidney deficiency with Du meridian obstruction.” The scale includes items such as dizziness, memory loss, physical weakness, insomnia, and soreness in the waist and knees. A 4-point Likert scale will be used, with higher scores indicating more severe symptoms. Evaluations will be conducted at weeks 0, 6, 8, and 12.

##### Evaluation Time Points

All outcome measures will be assessed at the following time points: baseline (week 0), midintervention (week 6), postintervention (week 8), and follow-up (week 12).

##### Data Management

CRFs will be completed on paper by data entry officers, and the data will be independently entered into a password-protected Microsoft Excel database by 2 trained personnel. Any discrepancies will be cross-checked to ensure accuracy. All trial documents—including informed consent forms, CRFs, and intervention records—will be securely stored in encrypted electronic databases and locked cabinets, under the supervision of the study coordinator. Adherence to the intervention (eg, attendance rate and missed sessions) will be documented in the CRFs and collected at weeks 0, 6, 8, and 12. Participant confidentiality will be maintained during result dissemination. At least one audit by the hospital’s ethics committee or research oversight body will be conducted to assess data completeness and regulatory compliance.

### Statistical Analysis

#### Overview

Data analysis will be conducted using SPSS (version 27.0; IBM Corp). The primary analysis will be based on the intention-to-treat principle, supplemented by per-protocol analysis. Missing data will be imputed using the last observation carried forward method. Results will be expressed as mean (SD) or median (IQR), as appropriate.

#### Primary Outcome Analysis

Analysis of the sole primary outcome is focused on the difference in MoCA scores at week 8 between the combined intervention group and the routine care group. We will use ANCOVA for this comparison, adjusting for the baseline MoCA score as a covariate, to derive the primary efficacy conclusion (significance level set at *P*<.05).

#### Secondary and Exploratory Analysis

All secondary outcomes (eg, Fried Frailty Phenotype, Barthel Index, and 36-item Short Form Health Survey), as well as all pairwise comparisons between the 4 intervention groups, will be considered exploratory analyses. Continuous variables will be tested for normality using the Shapiro-Wilk test. Normally distributed variables will be compared using 1-way ANOVA, followed by the Bonferroni correction for post hoc comparisons. Non-normally distributed variables will be analyzed using the Kruskal-Wallis test, followed by the Dunn test for pairwise comparisons. For changes over multiple time points (weeks 0, 6, 8, and 12), repeated measures ANOVA or the Friedman test (for nonnormal data) will be used for within-group and between-group comparisons over time. The categorical variables (eg, frailty status) will be analyzed using chi-square tests or the Fisher exact test.

#### Mechanism Verification and Interaction Test

To formally explore whether the combined therapy offers a synergistic effect (superior to the simple sum of the individual therapies), we will conduct a formal interaction analysis using a 2×2 factorial structure (Du-moxibustion factor×spine-pinching factor) within the ANCOVA framework. Furthermore, preplanned contrast analyses will be performed to test specific hypotheses, including the superiority of the combined intervention group over the individual Du-moxibustion group and the individual spine-pinching group. These results will also be treated as exploratory findings.

#### Strategy for Controlling Multiple Comparison Risk

To strictly control the risk of type I error inflation due to multiple comparisons, this study will only perform statistical inference and power evaluation on the primary outcome comparison. Formal multiple comparison adjustments will not be applied to the secondary outcomes or multitime-point comparisons. These results will be considered exploratory findings, used only to describe trends and generate future research hypotheses, and will be interpreted with extreme caution, not as definitive conclusions.

### Safety Monitoring

Adverse events will be monitored and recorded after each intervention session, including moxibustion burns, skin allergies, moxibustion syncope, and localized tenderness in the intervention groups, as well as psychological or emotional discomfort (eg, anxiety) in the control group. Adverse events will be classified by severity: mild (no treatment required), moderate (intervention paused; eg, cold compress for burns), and severe (intervention terminated and referral required; eg, hospitalization). The research nurse will document the time, symptoms, management, and outcomes in the CRF. Serious adverse events, such as hospitalization or life-threatening conditions, will be reported to the ethics committee within 24 hours. The DSMC will oversee trial safety and may trigger unblinding if necessary. Monitoring will cover the 8-week intervention and 12-week follow-up periods.

### Quality Assurance

Prior to participant enrollment, all research staff will undergo standardized training on trial implementation. This includes procedures for moxibustion and spinal pinching techniques, precautions, intervention frequency and duration, questionnaire administration and scoring, and outcome measurement protocols. The trial will adhere to principles of randomization and blinding. Randomization will be performed by noninvestigator personnel using statistical software, with strict allocation concealment to minimize selection bias. Participants and operators will remain blinded to group assignments to prevent contamination and ensure methodological rigor. A pilot trial will be conducted with four prefrail participants to refine intervention protocols. Data collection will avoid leading questions and ensure completeness. Double data entry will be used to prevent omissions and errors.

### Quality Control

Before the trial begins, all personnel, including operators, assessors, and data entry staff, will receive training on moxibustion and spinal pinching techniques, safety precautions, and the 8-week treatment schedule (1 session/wk for control group; 2 sessions/wk for intervention groups). Standardized assessments of MoCA and Fried frailty scores, as well as questionnaire distribution and verification, will be covered through both theoretical and practical sessions. Randomization and blinding protocols outlined in the “Randomization, Allocation, and Blinding” section will be followed. A noninvestigator will generate the randomization sequence using SAS 9.3 and perform allocation concealment. Participants will be unaware of their group assignments or intervention types. Although operators will not be blinded, they will not participate in assessments. A pilot study with 4 prefrail older participants will be conducted to refine the procedures (eg, frequency and technical steps). Data collection will avoid suggestive language, and questionnaires will be reviewed on-site. Double-checking by 2 personnel will ensure data quality. The DSMC and ethics committee will oversee trial quality through regular audits.

## Results

**Figure 4.**
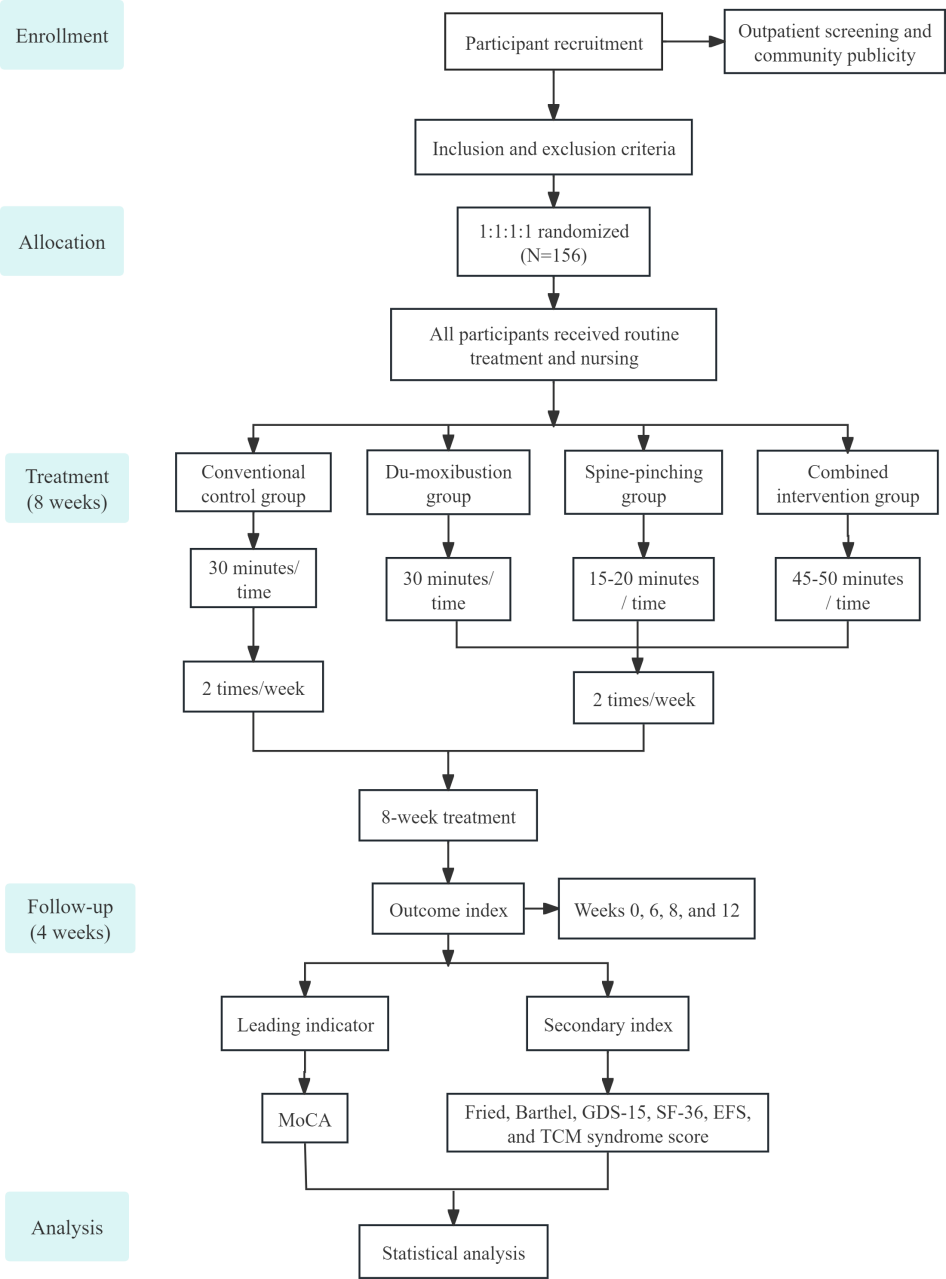
Trial flow diagram of the study. EFS: Edmonton Frail Scale; GDS-15: 15-item Geriatric Depression Scale; MoCA: Montreal Cognitive Assessment; SF-36: 36-item Short Form Health Survey; TCM: traditional Chinese medicine.

Recruitment for this study is scheduled to commence in March 2026 for eligible older participants. The 8-week intervention for the final participant is slated for completion in June 2027. All follow-up and data collection activities will be finalized by October 2027. Data analysis and the reporting of primary results are anticipated to be completed in the first quarter of 2028. A flow diagram of the trial process is shown in Figure 4.

## Discussion

This RCT is designed to evaluate the combined effects of Du-moxibustion and spinal pinching therapy on CF in prefrail older adults. By targeting both cognitive and physical decline at an early, potentially reversible stage, this study addresses a critical gap in the current evidence base, where few interventions simultaneously engage multidomain mechanisms relevant to CF [17]. Findings from this work are expected to contribute novel insights into integrative, nonpharmacological strategies for early prevention of cognitive deterioration.

CF—a clinical construct characterized by the coexistence of MCI and physical frailty—has been recognized as a precursor to dementia, disability, and mortality [18]. Despite increasing research interest, available treatments remain limited. Pharmacological therapies have shown only modest benefits for cognitive outcomes and carry risks of adverse effects [19], while exercise-based or nutritional interventions often exhibit suboptimal adherence and do not fully address the complex interplay between neurocognitive, musculoskeletal, and systemic vulnerability [20]. Consequently, there is a growing need for multidimensional, low-risk strategies suitable for early-stage intervention.

TCM offers a conceptual and therapeutic framework aligned with the multidomain nature of CF. According to the TCM theory, cognitive and physical decline in older adults frequently arises from spleen-kidney deficiency and impaired circulation in the Du meridian [21]. External therapies such as moxibustion and spinal pinching are believed to “warm yang,” “nourish the brain,” regulate gastrointestinal function, and restore systemic vitality [22]. Importantly, emerging biomedical evidence provides preliminary support for these mechanisms. Studies indicate that moxibustion may modulate neuroinflammatory pathways, enhance mitochondrial activity, promote neurotrophic factor expression, and regulate autonomic function [23]. At the peripheral level, moxibustion has been shown to improve gastrointestinal motility, reduce systemic inflammation, and enhance microcirculation—processes closely related to frailty progression [24].

Spinal-pinching therapy, though less extensively studied, has demonstrated potential neuromodulatory effects. Mechanistic studies suggest that stimulation along the paraspinal region may influence vagal tone, gastrointestinal-brain axis communication, and immune regulation [25]. These actions are consistent with improvements in appetite, digestion, and physical strength described in clinical practice. Therefore, combining Du-moxibustion and spinal pinching may produce synergistic effects by simultaneously engaging central regulatory pathways (eg, neuroendocrine-immune modulation) and peripheral support systems (eg, gastrointestinal function and muscular vitality) [26]. The principle of “unblocking before tonifying” further provides a rationale for integrating these therapies to enhance functional recovery in individuals with prefrailty.

The methodological design of this study strengthens its scientific rigor. The 4-arm randomized controlled design allows differentiation of the specific and additive effects of Du-moxibustion, spinal pinching therapy, and their combination—a level of comparative evidence rarely achieved in existing TCM research [27]. Standardized operating procedures, assessor blinding, clearly defined intervention parameters, and multidimensional outcome measures contribute to internal validity. The chosen treatment dose (30 min per session, twice weekly for 8 wk) aligns with parameters commonly used in moxibustion trials [28], ensuring sufficient thermal stimulation of the governor vessel while supporting participant adherence and safety. The inclusion of a 12-week posttreatment follow-up enables assessment of both immediate and sustained effects, addressing a common limitation in TCM clinical trials [29].

Nevertheless, several limitations must be acknowledged. First, complete participant blinding is infeasible due to the sensory characteristics of external TCM therapies, raising the possibility of expectation effects. Although assessor blinding mitigates this concern, residual bias cannot be entirely excluded. Second, the single-center nature of the trial may restrict the diversity of participants, limiting generalizability across different healthcare settings and cultural contexts. Third, stringent eligibility criteria requiring prefrailty, CF, and a specific TCM syndrome pattern (“spleen–kidney deficiency with Du meridian obstruction”) enhance internal validity but narrow the applicable population. Many older adults with cognitive or physical impairment may present with alternative syndrome patterns, and future studies should consider multiple TCM subtypes to broaden applicability.

In addition, although this study includes comprehensive functional and symptomatic assessments, biological indicators are absent. The lack of mechanistic biomarkers limits the ability to delineate physiological pathways underlying treatment effects. Incorporating inflammatory markers (eg, interleukin-6, tumor necrosis factor-α, and C-reactive protein), neurotrophic factors (eg, brain-derived neurotrophic factor), mitochondrial function indicators, or neuroimaging modalities (eg, functional magnetic resonance imaging, functional near-infrared spectroscopy, and electroencephalography) in future research may help clarify the neurobiological basis of TCM external therapies [30]. Multicenter studies with larger, more diverse samples and comparative evaluation of different syndrome patterns would further enhance generalizability and allow more robust conclusions regarding clinical applicability [31].

In conclusion, the integrated application of Du-moxibustion and spinal pinching therapy represents a promising, multidomain intervention for early management of CF. By combining central neuroregulatory effects with improvements in peripheral physiological function, this approach aligns with both TCM principles and emerging biomedical evidence [32]. Although limitations exist, the results of this trial may provide foundational evidence supporting scalable, culturally rooted, and mechanistically plausible strategies for promoting healthy aging and delaying cognitive and physical decline.

## Abbreviations

CF: cognitive frailty
CRF: case report form
DSMC: Data and Safety Monitoring Committee
MCI: mild cognitive impairment
MoCA: Montreal Cognitive Assessment
RCT: randomized controlled trial
SCI: Science Citation Index
TCM: traditional Chinese medicine
